# Effect of medical thoracoscopy‐guided intrapleural docetaxel therapy to manage malignant pleural effusion in patients with non‐small cell lung cancer: A pilot study

**DOI:** 10.1111/1759-7714.13158

**Published:** 2019-08-06

**Authors:** Myeong Geun Choi, Sojung Park, Dong Kyu Oh, Hyeong Ryul Kim, Geun Dong Lee, Jae Cheol Lee, Chang‐Min Choi, Wonjun Ji

**Affiliations:** ^1^ Department of Pulmonary and Critical Care Medicine, Asan Medical Center University of Ulsan College of Medicine Seoul South Korea; ^2^ Division of Pulmonary and Critical care medicine, Department of Internal Medicine Ewha Womans University College of Medicine Seoul South Korea; ^3^ Department of Thoracic and Cardiovascular Surgery Asan Medical Center Seoul South Korea; ^4^ Department of Oncology, Asan Medical Center University of Ulsan College of Medicine Seoul South Korea

**Keywords:** Docetaxel, malignant pleural effusion, pleurodesis

## Abstract

**Background:**

Although chemical pleurodesis is a useful treatment option for malignant pleural effusion, little is known about the effects of intrapleural docetaxel therapy.

**Objectives:**

This study aimed to evaluate the effects of medical thoracoscopy‐guided intrapleural docetaxel therapy in patients with lung cancer.

**Methods:**

Patients with lung cancer who diagnosed malignant pleural effusion were enrolled in this single‐center prospective pilot study. The clinical response and toxicity were evaluated at two, six and 12 weeks post‐treatment.

**Results:**

Medical thoracoscopy‐guided intrapleural docetaxel therapy was conducted in four patients between June 2016 and August 2017. The control rate of malignant pleural effusion was 100% (4/4), and the progression‐free duration of effusion was 527 ± 109 days. No serious adverse events were observed, but only mild‐to‐moderate adverse events were observed and well controlled by conservative management. Although the overall quality of life assessed using questionnaires did not show significant improvement, symptom burden due to dyspnea was significantly improved.

**Conclusions:**

Intrapleural docetaxel therapy with medical thoracoscopy showed good clinical responses, relieving dyspnea symptoms and providing tolerable safety profiles in patients with non‐small cell lung cancer (NSCLC) with malignant pleural effusion. A further prospective trial is warranted to evaluate the clinical effects of intrapleural docetaxel therapy in order to compare it with other treatment modalities.

## Key points

**Significant findings of the study**: Intrapleural docetaxel therapy with medical thoracoscopy showed good clinical responses, relieving dyspnea symptoms and providing tolerable safety profiles in patients with NSCLC‐related malignant pleural effusion.

**What this study adds**: This is the first study that evaluates one‐stage intrapleural docetaxel therapy with medical thoracoscopy. This approach may be a useful and safe palliative treatment modality in patients with malignant pleural effusion.

## Introduction

Malignant pleural effusion is a major cause of poor quality of life (QOL)^1^, ^2^ and survival^3^ in patients with advanced lung cancer. It is frequently aggravated in number, often symptomatic, and has a significant effect on decreasing the functional capacity and QOL. With malignant pleural effusion, these symptoms may result in reducing the performance status and make it difficult to perform systemic chemotherapy.^4^

Thoracentesis, chest tube insertion, pleurectomy, pleuroperitoneal shunt and pleurodesis using chemical agents such as sclerosing or antineoplastic, are the conventional treatment options for malignant pleural effusion.^5^ The most common treatment is chemical pleurodesis, using a chemical agent to induce pleural inflammation and adhesion by fibrin deposition, thereby preventing recurrence of pleural effusion and improving dyspnea. The chemical agent can be injected using a thoracoscope or intrapleural catheter, and the efficiency varies depending on the agent. To date, the safety and efficacy of chemical agents used in pleurodesis have been studied; however, comparable data were not available, and choosing the ideal agent remains controversial.^6^, ^7^, ^8^, ^9^ Talc, doxycycline and antineoplastic agents, such as docetaxel, paclitaxel and bleomycin, are used as chemical agents. Docetaxel is a taxane derivative that inhibits mitosis by blocking protein degradation and stabilizes by binding to microtubule, a protein component of tubulin. Therefore, it inhibits differentiation of cancer cells and makes cancer cells apoptotic and is one of the second‐line standard chemotherapies in non‐small cell lung cancer (NSCLC).^10^ In addition, docetaxel is known to be twice as potent as paclitaxel and acts at least five times more strongly in paclitaxel‐resistant cells.^11^

The efficacy of docetaxel as a systemic chemotherapeutic agent in advanced or metastatic NSCLC has already been confirmed; however, the effects and doses of intrapleural injection on malignant pleural effusion with lung cancer have only been reported in a Phase I study by Jones *et al*.^11^ In addition, the actual efficacy and effectiveness of intrapleural therapy and the use of medical thoracoscopy have not yet been reported. This pilot study aimed to evaluate the efficacy of therapy and prognosis in malignant pleural effusions by infusion docetaxel into the pleural cavity after removing the pleural adhesion with drainage of the pleural fluid using the medical thoracoscopy.

## Methods

### Study population

This was a prospective pilot study on patients diagnosed with NSCLC and those aged 20 and 80 years with symptomatic malignant pleural effusion. The inclusion criteria in this study were an Eastern Cooperative Oncology Group performance status of ≤2 and adequate hematological, renal and hepatic function. The exclusion criteria in this study were patients who underwent previous pleurodesis, thoracic radiosurgery, with bilateral pleural effusions, or with hypersensitivity reactions to docetaxel.

**Figure 1:**
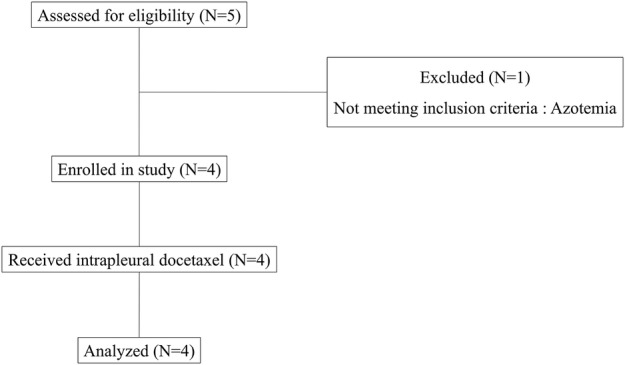
Consort flow diagram of the study.

As a pilot study, the crude number of subjects was calculated based on the study duration and estimated target population number. The consort flow diagram is described in Fig 1. All participants had provided their written informed consent. This study was approved by the institutional review board of the Asan Medical Center (IRB No. 2015–0118).

### Medical thoracoscopy procedure and intrapleural docetaxel therapy protocol

All patients underwent moderate sedation with midazolam and local anesthesia with lidocaine, followed by skin incision, soft tissue dilatation and insertion of a 21 mm trocar into the pleural cavity. Subsequently, a semi rigid medical thoracoscope (Olympus LTF‐260, Japan) was used to explore the pleural cavity and to remove the malignant pleural effusion. A 14‐Fr chest tube was inserted in the pleural cavity after the medical thoracoscopy. Docetaxel 75 mg/m^2^ was mixed with 100 mL 0.9% sodium chloride solution and slowly administered through the inserted chest tube. After the docetaxel injection, the tube was clamped for four hours and then declamped and drained. If no complications occurred, the chest tube was then removed and the patient was discharged the next day. A serial chest x‐ray was performed in all patients two weeks after docetaxel injection, and changes in malignant pleural effusion were evaluated.

### Measurement of clinical response and adverse events

The primary endpoint in this study was effusion control rate in medical thoracoscopy‐guided intrapleural docetaxel therapy. Safety profile and QOL changes were the secondary endpoints. Chest x‐ray and routine laboratories were performed two weeks post‐procedure. The treatment response was evaluated by comparing the post‐procedure x‐ray with baseline x‐ray. Changes in pleural fluids were evaluated by chest x‐ray at two, six, and 12 weeks post‐procedure in all participants. The response criteria used were based on a previous study by the Lung Cancer Study Group in a trial on intrapleural cisplatin and cytarabine in patients with malignant pleural effusion.^12^ Complete response (CR) was defined as complete disappearance of pleural effusion or decreased pleural effusion to the extent that thoracentesis was not possible. Partial response (PR) was defined as 75% decrease or more in pleural effusion, stabilization as <75% decrease or <25% increase in pleural effusion, and progression as 25% or more increase in pleural effusion. Effusion control rate was defined as the rate of patients showing response higher than the SD. All patients were examined for immediate adverse reactions at the first 24 hours initially and 7 ± 2 days post‐procedure. All adverse events were recorded according to the common terminology criteria for adverse events (CTCAE).^13^

### QOL assessment and adverse events

QOL and respiratory symptom assessment was performed using the QLQ‐LC13 questionnaire,^14^ which included questions on lung cancer‐associated symptoms (cough, hemoptysis, dyspnea and site‐specific pain) and treatment‐related side effects (sore mouth, dysphagia, peripheral neuropathy and alopecia). Three items were used to assess dyspnea. Every scale of each questionnaire was transformed to a 0 to 100 score as recommended in the EORTC scoring manual. The higher the score, more severe are the symptoms. Patients completed the questionnaire four times, at baseline and at days one, seven, and 21 post‐treatment.

### Statistical analysis

Data obtained from the participants were used to compare the demographic and baseline values among patients. The survival analysis was performed using the Kaplan‐Meier estimation method. Statistical analyses were performed using IBM SPSS version 23.0 (IBM Corp., Armonk, NY, USA).

## Results

### Patient baseline characteristics

Four patients diagnosed with lung cancer with malignant pleural effusion were enrolled from August 2016 to June 2017. All patients had pleural effusions unilaterally, characterized by exudate effusion. Malignant cells were found in the pleural fluid of three patients; however, the other one only showed few atypical lymphocytes. However, this lymphocyte dominant exudative pleural fluid could be considered as a malignant effusion by excluding tuberculosis and other infections. The mean age of patients was 58 (range 54–67) years, and half of the patients were men. Primary cancers in all four patients were adenocarcinoma, and two of them had epidermal growth factor receptor (EGFR) driver mutation. The ECOG performance status of all participants was two. One patient developed malignant pleural effusion in the disease progression state after the second treatment regimen, and three patients had malignant pleural effusion after the first treatment. The initially drained pleural fluid was approximately 700–1500 mL via a medical thoracoscope, and the chest tube was then inserted. Demographic information, tumor location, and pleural fluid characteristics are described in Table 1.

**Table 1:** Baseline characteristics of patients with non‐small cell lung cancer with malignant pleural effusion

	Patient 1	Patient 2	Patient 3	Patient 4
Age	57	54	67	54
Sex	F	M	M	F
Morphology	Adenocarcinoma	Adenocarcinoma	Adenocarcinoma	Adenocarcinoma
ECOG status	2	2	2	2
BSA (m^2^)	1.83	1.88	1.59	1.91
Smoking history	Never smoker	Former smoker	Never smoker	Never smoker
Smoking amount (Pack*years)		9		
Discontinuation period (years)		17		
Pain scale (VAS)	3	1	3	4
mMRC grade	2	3	2	2
The previous treatment before MPE†	Second line	First line	First line	First line
EGFR mutation	+	+	−	−
Amounts of drained pleural effusion before chest tube insertion (mL)	1300	1500	1500	720
Pleural fluid analysis				
pH	7.2	7.8	7.6	7.8
RBC (/μL)	48 640	98 750	900	5000
WBC (/μL)	1000	1100	2200	960
Neutrophils (%)	3	4	0	1
Lymphocytes (%)	15	54	61	93
Histiocytes (%)	51	6	7	4
Eosinophils (%)	18	7	0	0
Malignant cells (%)	8	28	27	0
Protein (g/dL)	5.5	5.4	4.9	5.2
Albumin (g/dL)	3.1	2.7	3	2.8
Glucose (mg/dL)	110	77	83	118
LD (U/L)	180	319	231	282
ADA (IU/L)	27.2	23.5	15.5	18.1

† Patient 1: Gefitinib > Pemetrexed; Patient 2: Gefitinib; Patient 3: Pemetrexed/Cisplatin; Patient 4: Pemetrexed/Cisplatin.

MPE, malignant pleural effusion.

### Clinical response of intrapleural docetaxel therapy

**Figure 2:**
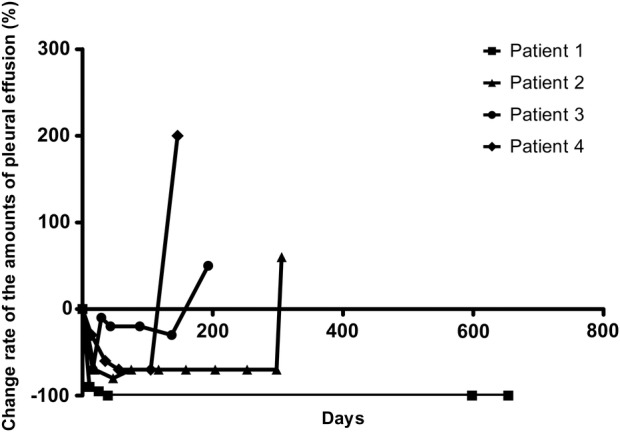
Overall response curve of intrapleural docetaxel therapy.

**Figure 3:**
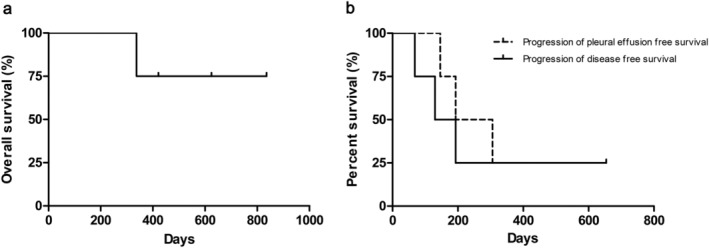
(**a**) Overall survival curve of patients with non‐small cell lung cancer with malignant pleural effusion. (**b**) Progression of disease free survival and progression of effusion free survival.

The effusion control rate was 100% (4/4). Three patients (75%) showed PR based on the malignant pleural effusion on chest x‐ray at two weeks post‐procedure, and one patient had SD (25%; Table 2). Malignant pleural effusions of three patients progressed at five, six and 10 months follow‐up, and one patient maintained CR more than 21 months (Table 3, Fig 2). One patient died during the study period; however, the cause of death remained unknown because the patient transferred to another hospital for supportive care (Fig 3). The median progression‐free survival of primary cancer was 161.5 (range 68–654) days, and the progression‐free duration of the pleural effusion was 249.5 (range 146–654) days (Table 3, Fig 3). In addition, the mean progression‐free duration of the pleural effusion in EGFR mutant lung cancer was 480 (range 306–654) days, while those with EGFR wild type lung cancer was 169.5 (range 146–193) days (Table 3).

**Table 2:** Clinical response of malignant pleural effusion after intrapleural docetaxel therapy

	Patient 1	Patient 2	Patient 3	Patient 4
2 weeks	PR	PR	PR	SD
6 weeks	CR	PR	SD	PR
12 weeks	CR	PR	SD	PR

**Table 3:** Time to pleural effusion and disease progression

	Patient 1	Patient 2	Patient 3	Patient 4	Mean	Median
Time to progression of pleural effusion (days)	654	306	193	146	324.75	249.5
Time to progression of disease (days)	654	68	193	130	261.25	161.5

### Safety profile and adverse events of intrapleural docetaxel therapy

Intrapleural docetaxel therapy did not result in serious adverse events that could lead to death. Two patients (50%) had mild subcutaneous emphysema, two (50%) had chest wall pain, two (50%) had pneumothorax, one (25%) had pleural effusion, one (25%) had anemia, and one (25%) had allergic reaction as adverse events; however, these symptoms were controlled by conservative treatments (Table 4).

**Table 4:** Adverse events of intrapleural docetaxel therapy

	Patient 1	Patient 2	Patient 3	Patient 4
Adverse events (Grade)				
Hematologic				
Neutropenia	‐	‐	‐	‐
Thrombocytopenia	‐	‐	‐	‐
Anemia	‐	‐	‐	2
Pulmonary				
Cough	‐	‐	‐	‐
Pneumothorax	‐	‐	1	1
Subcutaneous emphysema	1	‐	1	‐
Dyspnea	‐	‐	‐	‐
Gastrointestinal				
Nausea	1	‐	‐	‐
Vomiting	‐	‐	‐	‐
Hepatic	‐	‐	‐	‐
Cardiovascular	‐	‐	‐	‐
Renal	‐	‐	‐	‐
Metabolic	‐	‐	‐	‐
Constitutional				
Fever	‐	‐	‐	‐
Fatigue	‐	‐	‐	‐
Anorexia	‐	‐	‐	‐
Chest and/or shoulder pain	1	1	‐	‐
Allergic reaction	‐	‐	‐	1

### QOL and respiratory symptoms

All four patients completed the QLQ‐LC13 questionnaire four times, and D1, D7 and D21 questionnaire were administered before knowing any results. Dyspnea items in the symptom scale showed a significant decrease between baseline and 21 days, with mean changes of −30.55 (CI 95%; −52.80, −8.31; *P* = 0.022). However, the lung cancer‐related symptom scale at baseline was 27.09 out of 100 points, and these scores showed a decreasing trend to 10.42 at D21 day, but were not statistically significant (*P* = 0.073). The total scale at baseline was 19.16 and 6.95 points at D21 day. Changes in the total QOL score were moderately significant (*P* = 0.057; Table 5).

**Table 5:** Quality of life and symptoms of patients and lung cancer module QLQ‐LC13 analysis

	Symptoms scale†			Dyspnea			Total scale‡		
	Mean	Interval Change (95% CI)	*P*‐value	Mean	Interval change (95% CI)	*P*‐value	Mean	Interval change (95% CI)	*P*‐value
Baseline	27.09	‐	‐	36.11	‐	‐	19.16	‐	‐
D1	23.96	−3.13 (−9.47, +3.22)	0.215	25.99	−11.11 (−36.11, +13.89)	0.252	17.08	−2.08 (−6.31, +2.15)	0.215
D7	20.83	−6.25 (−28.24, +15.74)	0.432	22.22	−13.89 (−44.08, +16.30)	0.239	14.99	−4.17 (−21.30, +12.97)	0.496
D21	10.42	−16.67 (−36.19, +2.85)	0.073	5.56	−30.55 (−52.80, −8.31)	0.022	6.95	−12.21 (−25.07, +0.65)	0.057

† Cough, hemoptysis, dyspnea, and site‐specific pain.

‡ Lung cancer‐associated symptoms (cough, hemoptysis, dyspnea, and site‐specific pain) and treatment‐related side effects (sore mouth, dysphagia, peripheral neuropathy, and alopecia).

## Discussion

A medical thoracoscopy‐guided intrapleural docetaxel therapy was a useful and relatively safe palliative treatment modality in patients with NSCLC associated with malignant pleural effusion. The control rate of malignant pleural effusion was 100% in this study. Regarding safety, adverse effects were mild and well controlled in most cases. In addition, the QOL of all patients improved, especially the dyspnea symptom that significantly decreased. This is the first study to analyze the therapeutic effects of medical thoracoscopy‐guided intrapleural docetaxel therapy in the actual clinical practice and to identify the palliative role of intrapleural docetaxel therapy that can improve QOL.

A previous Phase II study on patients with malignant pleural effusion administer intrapleural paclitaxel reported 92.9% overall response rate at four weeks.^15^ Besides, a Phase I study using intrapleural docetaxel showed 90% overall response rate after three weeks of treatment.^11^ In this study, the overall response rate, the primary endpoint, was 75% at two weeks post‐procedure, and the effusion control rate was 100%. Although previous studies showed higher overall response rates of malignant pleural effusion than the current study at three or four weeks, it seemed to be due to the different time points on the effect estimation and because of the small sample size in this study. Regarding the overall response rate at six weeks, this study showed 100% because the patient who did not respond at two weeks showed PR. This finding was similar with those of the previous studies.

Malignant pleural effusion was observed with an increase in tumor burden during the first‐line or second‐line systemic treatment in all four patients. All four patients complained of severe dyspnea; therefore, intrapleural therapy was performed before the next‐line systemic chemotherapy to relieve the symptoms. After 2–11 weeks of intrapleural therapy, systemic chemotherapy was performed, while the control rate of malignant pleural effusion was 100%. In particular, patients 1 and 4 started systemic chemotherapy five and three weeks after intrapleural therapy, respectively, because they had to receive palliative radiation therapy for brain or bone metastasis. Interestingly, patient 2 maintained the first‐line treatment of EGFR tyrosine kinase inhibitor according to the beyond progression treatment strategy.^16^ Therefore, we were able to estimate the immediate effect of intrapleural therapy on malignant pleural effusion without the effects of systemic treatment. Furthermore, this study evaluated a longer response to intrapleural docetaxel treatment, unlike those of the previous studies, which showed short‐term responses at 3–4 weeks,^11^, ^15^ and confirmed how malignant pleural effusion changes with disease progression. The progression‐free survival of the pleural effusion was 527 days, whereas that of the disease was 479 days. The progression of malignant pleural effusion was not observed for a certain period of time. Interestingly, pleural effusion in patients with EGFR mutant lung cancer was found to be controlled longer, and patients with good response within 12 weeks had delayed progression of pleural effusion after disease progression. However, this is not conclusive because EGFR mutant lung cancer generally showed better treatment response to EGFR tyrosine kinase inhibitors and survival. Therefore, further prospective studies should be conducted to confirm the difference of treatment effects according to mutation status.

A significant improvement in dyspnea was found during the QOL assessment through questionnaires, and this was consistent with that of the previous study on thoracoscopic pleurodesis.^17^ All patients experienced improved QOL, although overall QOL improvement was not statistically significant, which is thought to be due to the small number of patients. Adequate palliative treatment is well known to improve survival in patients with advanced lung cancer.^18^ Thus, improving the QOL and alleviating symptoms are important when managing a patient with advanced lung cancer. In this manner, intrapleural docetaxel therapy could have a palliative role in patients with lung cancer associated with malignant pleural effusion.

This was the first study conducted on a one‐stage intrapleural docetaxel therapy with medical thoracoscopy guidance. Previous studies have only performed thoracostomy via the chest tube and chemical pleurodesis sequentially after tube drainage.^6^, ^11^, ^15^ Conversely, medical thoracoscopy was performed to evaluate the pleural cavity and to drain the malignant pleural effusion as much as possible. After draining all the effusion, intrapleural docetaxel therapy was immediately performed after placing the chest tube. This method reduced the waiting time for drainage, the possibility of insufficient drain and the length of hospital stay.

Adverse events of intrapleural docetaxel were similar to those of previous studies.^6^, ^11^, ^15^ Subcutaneous emphysema, chest wall pain, pneumothorax, anemia and allergic reactions were reported as adverse effects, but all were mild to grade 1–2, and no serious adverse events were reported. Based on the result, intrapleural docetaxel treatment was suggested as a relatively safe treatment modality. In particular, chest wall pain and pneumothorax were the most common symptoms; therefore, sufficient pain control would be required, and the presence of pneumothorax should be determined through chest x‐ray.

A few limitations should be considered in this study. Firstly, this study was conducted in only four patients with NSCLC because the purpose of this pilot study was to evaluate the effects of intrapleural docetaxel therapy. Although medical thoracoscopy‐guided intrapleural docetaxel therapy was found to be a useful and relatively safe palliative treatment modality in patients with NSCLC‐related malignant pleural effusion, further large‐scale studies are warranted to confirm this treatment effect. Secondly, the treatment effect was not compared to other treatment modalities, such as talc or other chemical agents. Further case‐control studies will be needed to investigate whether the effectiveness of treatment is caused by pleural effusion drainage or pleurodesis itself, and whether docetaxel is more effective than other chemical pleurodesis agents. Thirdly, it did not show a significant QOL improvement, although the participants’ overall QOL showed an improved pattern. However, dyspnea was significantly improved by intrapleural docetaxel therapy. This low statistical power was considered to be caused by the small sample size. Therefore, a large‐scale study is warranted. Finally, only patients diagnosed with adenocarcinoma were included in this study. Although adenocarcinoma is reported as the most common histologic type of lung cancer in South Korea,^19^ further evaluation is needed to determine whether this procedure will be effective in other cell types of lung cancer. The systemic antitumor effect of intrapleural docetaxel therapy, such as disease progression or mortality, also may need to be studied with a longer study period.

## Conclusion

Intrapleural docetaxel therapy using a medical thoracoscopy showed good clinical responses, relieving dyspnea symptoms and providing tolerable safety profiles in patients with NSCLC‐related malignant pleural effusion. In conclusion, medical thoracoscopy‐guided intrapleural docetaxel therapy is suggested as a useful and safe palliative treatment modality in patients with malignant pleural effusion. In the future, large‐scale prospective clinical studies with control groups are required to confirm the efficacy of intrapleural docetaxel therapy by comparing with other treatment modalities.

## Disclosure

This study was financially supported by a grant from Boryung Pharmaceutical Co., Ltd. The funders had no role in the writing of the manuscript or the decision to submit it for publication.

The authors have no conflicts of interest to declare.
